# Mercury Exposure and Associations with Hyperlipidemia and Elevated Liver Enzymes: A Nationwide Cross-Sectional Survey

**DOI:** 10.3390/toxics8030047

**Published:** 2020-07-01

**Authors:** Seungho Lee, Sung-Ran Cho, Inchul Jeong, Jae Bum Park, Mi-Yeon Shin, Sungkyoon Kim, Jin Hee Kim

**Affiliations:** 1 Department of Occupational & Environmental Medicine, Ajou University School of Medicine, Suwon 16499, Korea; lgydr@aumc.ac.kr (S.L.); icjeong0101@aumc.ac.kr (I.J.); jbpark@ajou.ac.kr (J.B.P.); 2 Department of Laboratory Medicine, Ajou University School of Medicine, Suwon 16499, Korea; sungran@aumc.ac.kr; 3 Department of Environmental Health Sciences, Graduate School of Public Health, Seoul National University, Seoul 08826, Korea; damage7@snu.ac.kr; 4 Department of Integrative Bioscience & Biotechnology, Sejong University, Seoul 05006, Korea

**Keywords:** mercury, obesogen, lipid profiles, hyperlipidemia, elevated liver enzymes

## Abstract

Mercury (Hg) has obesogenic properties. However, the associated health outcomes of population-level mercury exposure were unclear. This study investigated the relationships between blood mercury levels and obesity-related outcomes such as hyperlipidemia and elevated liver enzymes. Using the second cycle of the Korean National Environmental Health Survey (*n* = 6454), we performed logistic regression to examine the effects of Hg on hyperlipidemia and elevated liver enzymes. The blood mercury levels were significantly higher in the hyperlipidemia group (*n* = 3699, male: 4.03 μg/L, female: 2.83 μg/L) compared to the non-hyperlipidemia group (*n* = 2755, male: 3.48 μg/L, female: 2.69 μg/L), and high blood mercury levels were associated with an 11% higher risk of hyperlipidemia. The elevated liver enzymes group had higher mean blood mercury levels (*n* = 1189, male: 4.38 μg/L, female: 3.25 μg/L) than the normal group (*n* = 5265, male: 3.64 μg/L, female: 2.70 μg/L), and elevated blood mercury was associated with a 35% higher risk of elevated liver enzymes. Moreover, the effect was constant after adjusting for personal medications. These results indicate that mercury exposure is significantly associated with hyperlipidemia and elevated liver enzymes.

## 1. Introduction

Obesity is a major risk factor for several chronic diseases, including hypertension, diabetes, and hyperlipidemia, and is a growing concern worldwide. The prevalence of metabolic syndromes in Korea is approximately 30% due to increasing obesity [1]. A Westernized diet, lifestyle patterns, and exposure to environmental pollutants are involved in the development of obesity. Endocrine-disrupting chemicals such as phthalates, phenols, polychlorinated biphenyl (PCBs), and polybrominated diphenyl ether (PBDEs) are well-known obesogens [2], and several studies have reported that mercury (Hg) is also associated with metabolic syndromes [3,4].

According to the first cycle of the Korean National Environmental Health Survey [5], the geometric mean (GM) of blood Hg among Koreans was 3.08 μg/L, which is high compared to the US (mean: 0.68 μg/L) [6] and Canada (mean: 0.59 μg/L) [7]. Blood Hg levels in Korea have been decreasing for the last ten years, but approximately 25% of the Korean population still has high levels over 5.00 μg/L, which represents the control value for blood Hg (HBM-I) [8]. 

The main reason for high blood Hg levels in the Korean population is frequent seafood consumption, due to the country’s geographical characteristics [9]. A previous study reported that methyl mercury (MeHg) exposure is approaching the reference dose within the Korean population, which is the allowable daily intake [10]. 

Exposure to Hg induces oxidative stress, lipid peroxidation, and mitochondrial dysfunction [11,12], and γ-glutamyltransferase (GGT), a well-known biological marker of oxidative stress, is significantly associated with blood Hg [13]. Interestingly, GGT levels may reflect insulin resistance [14] and cardiovascular risk because of the relationship with lipoprotein cholesterol oxidation [15]. In a cohort study from Japan, the risk of metabolic syndrome and diabetes increased with the levels of hepatic enzymes, such as alanine aminotransferase (ALT), aspartate aminotransferase (AST), and GGT, among the metabolic syndrome–free participants [16]. Thus, the hepatic enzymes may serve as surrogate markers of obesity.

Several studies have investigated the associations between Hg and health, but the population-level health effects of Hg exposure remain unclear. Therefore, we hypothesized that Hg exposure induces obesity-related outcomes and investigated the relationships between blood Hg and hyperlipidemia and elevated liver enzymes. We used national biomonitoring data to identify the variables that influence blood Hg and analyzed the relationships between blood Hg and the lipid profiles and hepatic enzymes. Finally, we assessed the effects of Hg on hyperlipidemia and elevated liver enzymes.

## 2. Material and Methods

### 2.1. Survey Data

The Korean National Environmental Health Survey (KoNEHS) is a nationwide cross-sectional biomonitoring survey that aims to monitor the trends of environmental chemicals, including blood Hg, and to identify major exposure sources. Approximately 2000 subjects (≥19 years) were annually recruited via stratified multistage sampling units to represent the residential distributions of geographical area, sex, and age. A total of 6454 participants provided blood and urine samples and questionnaire responses, including demographic information and lifestyle. The KoNEHS was approved by the Research Ethics Committee of the National Institute of Environmental Research (NIER #2014-01-01-074, date of approval: 20 March 2014), Korea. Written informed consents were obtained from all participants. Detailed study information is provided in a prior publication [17]. The present study used data from the second cycle of the KoNEHS, conducted between 2012 and 2014. 

### 2.2. Measurement

The participant blood samples were collected in EDTA-containing tubes. After mixing, the blood samples were aliquoted into cryo-tubes and stored at −20 °C. The blood chemistry markers were measured by Seoul Clinical Laboratories (SCL, Yongin, South Korea), with a reference laboratory service [18]. Briefly, the serum concentrations of total cholesterol, high-density lipoprotein (HDL) cholesterol, and triglycerides (TG) were measured by an enzymatic method using auto analyzer ADVIA 1800 (Siemens Medical Solutions, USA). Serum low-density lipoprotein (LDL) cholesterol concentrations were calculated from Friedewald’s equation [19]. Calculated LDL values less than zero were designated as 0 (*n* = 37). The hepatic enzymes (ALT, AST, and GGT) were measured on an auto-analyzer ADVIA 1800 (Siemens Medical Solutions, Malvern, PA, USA). 

Total Hg was measured by flow injection cold-vapor atomic absorption spectrometry (DMA 80, Milestone, Bergamo, Italy) using whole blood samples. The limit of detection (LOD) for blood Hg was 0.10 μg/L. A value below the LOD (*n* = 1) was included as LOD divided by the square root of 2. External quality control was performed twice per year by the Korean Association of Quality Assurance for Clinical Laboratory (KSLM) and the German External Quality Assessment Scheme for analysis of heavy metals in biological materials (G-EQUAS) [17].

### 2.3. Criteria for Hyperlipidemia and Definition of the Elevated Liver Enzymes

The criteria for hyperlipidemia were taken from the National Cholesterol Education Program—Adult Treatment Panel III (NCEP-ATP III) [20]. Based on these guidelines, hyperlipidemia was defined as lipid profiles showing high LDL (above 130 mg/dL), high total cholesterol (above 200 mg/dL), or high triglycerides (above 150 mg/dL). Elevated liver enzymes were defined by the reference ranges provided by SCL; ALT concentrations above 49 U/L, AST concentrations above 34 U/L, or GGT concentrations above 73 U/L (for men, above 38 U/L for women) [21].

### 2.4. Statistical Analyses

Subjects with missing records of blood Hg, lipid profiles, and hepatic enzymes were excluded (*n* = 24). The final dataset contained 6454 personal records, and the distribution of blood Hg was calculated using sampling weights and survey strata information. The blood Hg distribution was right-skewed, so a log-transformation was performed to satisfy the assumptions of normality. Bivariate analyses were initially performed to evaluate the demographic variables, including sex (male, female), age group (19–29, 30–39, 40–49, 50–59, 60–69, and >70), BMI (underweight: <18.5, normal: 18.5–23, overweight: 23–25, and obese: >25), smoking status (non-smoker, past-smoker, or current smoker), alcohol consumption frequency (never, <1 time/month, 1–3 times/month, 1–2 times/week, >3 times/week, or daily), household monthly income (<USD 1500, USD 1500–USD 3000, USD 3000–USD 5000, USD 5000–USD 10000, and ≥USD 10000), and fish consumption (rarely, 1–3 times/month, 1–3 times/week, or 4–6 times/week). We divided the blood Hg levels into three groups based on the interquartile range, low (blood Hg < 25^th^), middle (25^th^ ≤ blood Hg < 75^th^), high (blood Hg ≥ 75^th^), and compared the blood lipid levels and hepatic enzymes among groups. Each marker was regressed on blood Hg with sex, age, BMI, smoking status, alcohol frequency, and income using sampling weights and survey strata information. Analysis of variance (ANOVA) by sex and analysis of covariance (ANCOVA) with age were used to assess the associations between blood Hg and criteria status of each clinical chemistry marker.

Logistic regression analyses were performed to examine the effect of Hg on hyperlipidemia and elevated liver enzymes. Self-reported personal medications were considered to adjust for the effect of medicine and individual health status. The corresponding health question was open-ended, so we extracted information for hyperlipidemia-associated diseases by including the following terms: ‘hyperlipidemia’, ‘dyslipidemia’, ‘high blood pressure’, ‘hypertension’, and ‘diabetes’. The terms ‘fatty liver’, ‘hepatitis’, ‘liver cirrhosis’, ‘liver disease’, and ‘elevated liver enzymes’ were included to represent personal medications for liver diseases. The final models were selected via model fit scores, such as the Akaike information criteria (AIC) and Bayesian information criteria (BIC). The main effects of sex, age, BMI, smoking status, alcohol frequency, and fish consumption, and the two-way interaction of sex and alcohol frequency were included in the final model with the Hg levels. Personal medication information was included in the logistic regression model as a covariate. Finally, the correlations between liver enzymes and lipid profiles were analyzed across the blood Hg groups as part of the sensitivity analysis. The significance level (alpha) was set to 0.05, and all of the statistical analyses were performed in SAS version 9.4 (SAS Institute Inc., Cary, NC, USA, 2013).

## 3. Results

### 3.1. The Distribution of Blood Hg

The geometric mean (GM) and 95^th^ percentile of blood Hg among all participants were 3.11 μg/L and 9.01 μg/L, respectively. The blood Hg levels were significantly higher in males (GM = 3.70 μg/L) than in females (GM = 2.63 μg/L; Table 1). Blood Hg levels increased until the participants were in their 60s. The blood Hg levels also increased as the BMI, alcohol frequency, and household income increased. Fish consumption > 4 times per week was associated with blood Hg levels that were approximately twice as high as those who rarely ate fish (GM = 4.04 μg/L vs. GM = 2.17 μg/L). Smoking, alcohol consumption frequency and amount, cooking types, education, marital status, parity, and menopause were also significantly related to blood HG, but herbal medicine had no influence (Supplementary Materials Table S1).

### 3.2. The Distribution of Lipid Profiles and Hepatic Enzymes

The blood Hg levels were categorized into three groups—low: ≤2.36 μg/L, medium: 2.36 < Hg ≤ 4.07 μg/L, and high: >4.07 μg/L. Approximately 45% of men and 24.6% of women were in the high blood Hg group. Table 2 shows the distribution of each marker across the blood Hg groups. The GMs of LDL, total cholesterol, and TG increased with blood Hg and was highest in the high blood Hg group for both sexes. HDL tended to decrease with increasing blood Hg in all populations, but the trend disappeared after stratifying by sex. The GMs of hepatic enzymes increased with blood Hg in both sexes, and blood Hg had a significant effect on all of the markers, except TG.

### 3.3. Associations between the Blood Hg Levels and Lipid Profiles

Table 3 shows the GMs of the blood Hg levels and their associations with the lipid profiles. Blood Hg increased until LDL levels reached ‘Borderline high’. Total cholesterol consistently increased with blood Hg in females, but the blood Hg levels decreased with a ‘High’ total cholesterol classification in males. The blood Hg levels significantly differed for each lipid profile after being adjusted for sex. However, the significant difference for HDL disappeared after considering age. According to the definitions for hyperlipidemia (LDL ≥ 130 mg/dL, total cholesterol ≥ 200 mg/dL, or TG ≥ 150 mg/dL), 61.8% of males (*n* = 1710) and 53.9% of females (*n* = 1989) were hyperlipidemia. The blood Hg levels were significantly higher in the hyperlipidemia group (male: 4.03 μg/L, female: 2.83 μg/L) compared to the non-hyperlipidemia group (male: 3.48 μg/L, female: 2.69 μg/L). 

### 3.4. Association between Blood Hg and the Hepatic Enzymes

The blood Hg levels were higher in participants who fell outside of the reference range for the hepatic enzymes (Table 4). The levels differed significantly by sex, and the significance remained after adjustment for age. According to the criteria for elevated liver enzymes, 24.3% of males (*n* = 671; ALT > 49 U/L, AST ≥ 34 U/L, or GGT ≥ 73) and 14.0 % of females (*n* = 518; ALT > 49 U/L, AST ≥ 34 U/L, or GGT ≥ 38) had elevated liver enzymes. The blood Hg levels were significantly higher in the elevated liver enzymes group (male: 4.36 μg/L, female: 3.25 μg/L) compared to the normal liver enzymes group (male: 3.64 μg/L, female: 2.70 μg/L).

### 3.5. The Risks of Hyperlipidemia and Elevated Liver Enzymes

**Figure 1:**
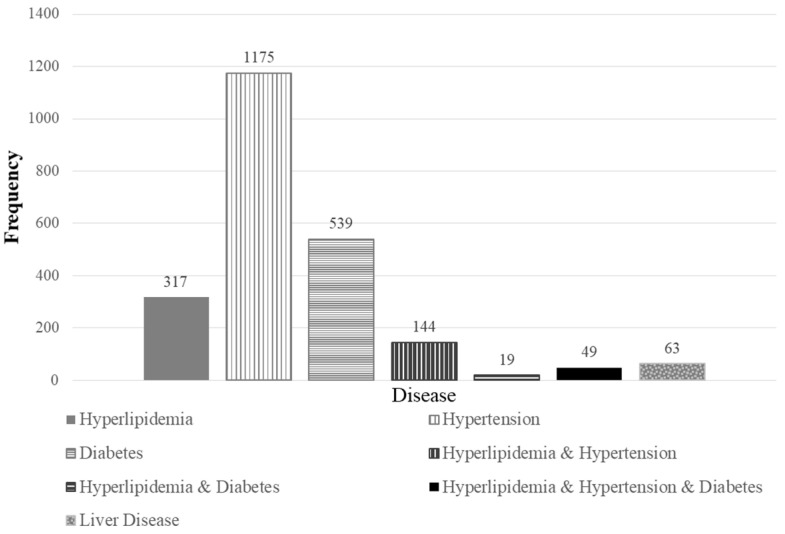
Frequency of the reported personal medications. Hyperlipidemia-associated diseases were extracted with the terms ‘hyperlipidemia’, ‘dyslipidemia’, ‘high blood pressure’, ‘hypertension’, and ‘diabetes’. Liver diseases were categorized with the terms ‘fatty liver’, ‘hepatitis’, ‘liver cirrhosis’, ‘liver disease’, and ‘elevated liver enzymes’.

Figure 1 shows the number of participants reporting personal medications; 317 (4.91%) reported hyperlipidemia, 1175 (18.2%) reported hypertension, and 539 (8.35%) reported diabetes. One hundred and forty-four subjects took medication for hyperlipidemia and hypertension, both. And 49 subjects indicated that they took medication for hyperlipidemia, hypertension, and diabetes. Increased blood Hg was associated with a 1.105-fold increase in the odds of hyperlipidemia (95% CI: 1.013, 1.208) (Table 5). Thus, an increase of 1 μg/L blood Hg was associated with an 11 % risk of hyperlipidemia. The significance of the odds ratio (OR) estimates remained even after adjustment for personal medications related to hyperlipidemia. For those participants reporting hyperlipidemia and diabetes, the blood Hg GM was 3.60 μg/L, and blood Hg was associated with a 1.105-fold increase in the odds of hyperlipidemia (95% CI: 1.013, 1.207). This remained after adjusting for personal medications (hyperlipidemia and diabetes).

Sixty-three (0.98%) participants reported fatty liver, hepatitis, cirrhosis, liver disease, increased hepatic enzymes, or other liver-related diseases. Increased blood Hg induced a 1.345-fold increase in the odds of elevated liver enzymes (95% CI: 1.206, 1.500). Thus, high blood Hg induced a 35% greater odds of elevated liver enzymes. After adjustment for personal medications related to liver diseases, the OR showed a 1.350-fold risk of elevated liver enzymes.

### 3.6. Relationships between the Lipid Profiles and Hepatic Enzymes across Blood Hg Groups

**Figure 2:**
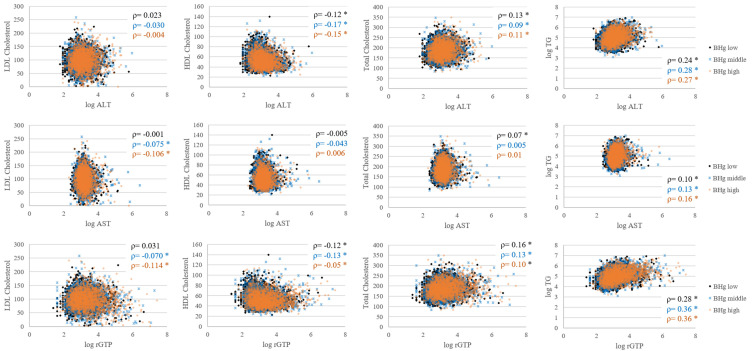
Correlations between the lipid profiles and hepatic enzymes in each blood Hg group. The x-axes represent the natural log scale of hepatic enzymes, and the y-axes represent the lipid profiles including natural log scale of TG. The black, blue, and orange colors indicate low, middle, and high blood Hg levels, respectively. ‘ρ’ represents the correlation coefficient, and * indicates the significance of the correlation coefficient (*p* < 0.05). BHg on the left side of the figure indicates blood Hg.

The correlations between the lipid profiles and hepatic enzymes in each blood Hg group are presented in Figure 2. In general, the correlation coefficients showed no associations between the hepatic enzymes and lipid profiles, except for TG. The correlation coefficients between log TG and log hepatic enzymes were 0.28 for ALT, 0.14 for AST, and 0.35 for GGT, and did not differ across blood Hg levels. 

## 4. Discussion

This study used the second cycle of the KoNEHS (2012–2014) to examine the obesogenic properties of blood Hg as it relates to hyperlipidemia and elevated liver enzymes. The GM of blood Hg was high, up to 3.11 μg/L, and 57.3% of the survey population had hyperlipidemia. For participants aged 40 and above, 55–66% had hyperlipidemia, whereas 38% of the participants in their 20s and 50 % of the participants in their 30s had hyperlipidemia. The mean BMI of the hyperlipidemia group was 25.0 compared to 23.3 in the non-hyperlipidemia group. Moreover, the mean BMI was 24.0 in the normal group and 25.7 in the elevated liver enzymes group. 

Approximately 32.1% of males (*n* = 889) and 15.4 % of females (*n* = 566) had blood Hg levels over 5.00 μg/L, which is the acceptable level for no adverse effects (HBM-I) [22]. These results are consistent with the first cycle of the KoNEHS (2009–2011), where 33.4% of males and 16.1% of females exceeded the HBM-I [10]. Many studies have investigated the high blood Hg levels in the Korean population. Some reported significant associations between Hg, high BMI, and metabolic syndromes [23,24], while others reported no associations or even negative associations [25,26,27]. Metabolic syndromes are associated with many factors, including dietary habits and living patterns, and some factors may have a stronger influence than Hg exposure. For example, alcohol consumption is a major risk factor for metabolic syndromes, and raw-fish and clam soup are popular menu items that are often consumed with alcohol in Korea. Thus, blood Hg is also significantly associated with drinking alcohol, and the frequency of alcohol intake should be accounted for when evaluating the relationship between Hg exposure and metabolic syndromes in Korea. In our study, 62.3% of males and 20.9% of females drank alcohol more than once a week. Therefore, we also included the interaction of alcohol consumption frequency and sex (*p* < 0.0001 for hyperlipidemia) when examining the obesogenic effects of Hg.

It is also possible that individual treatments for obesity attenuate the effects of Hg exposure. Those diagnosed with metabolic syndromes may actively control their lipid profiles and insulin resistance. Therefore, treatments including personal medications, could affect the associated markers and the diagnoses of metabolic syndromes. In the Table 5, we showed only the ORs of blood Hg after each medication. And the same analyses provided that taking personal medications for hyperlipidemia reduced the odds of hyperlipidemia by 29% (OR: 0.710, 95% CI: 0.559, 0.902), and taking personal medications for hyperlipidemia, hypertension, and diabetes was associated with 59% lower odds of hyperlipidemia (OR: 0.410, 95% CI: 0.029, 0.733). Nonetheless, Hg significantly affected to the odds of having hyperlipidemia.

Among the different forms of Hg, alkyl Hg is more lipid soluble and passes readily through biological membranes [28]. Especially, methylmercury (MeHg) among the alkyl Hg, is the dominant form in human blood [29] because the primary exposure source for the general population is fish consumption. MeHg exposure inhibits paraoxonase-1, which prevents the atherosclerotic process by metabolizing toxic oxidized lipids associated with LDL and HDL [30]. Therefore, Hg induces oxidative stress and disrupts gluconeogenesis, resulting in systemic inflammation that affects the accumulation of abnormal adipocytes [23,31]. Our results showed that the levels of blood Hg were significantly higher (*p* <.0001) in the hyperlipidemia group (male: 4.03 μg/L, female: 2.83 μg/L) than in the non-hyperlipidemia group (male: 3.48 μg/L, female: 2.69 μg/L), and that an increase of 1 μg/L blood Hg was associated with an 11% increase in the odds of hyperlipidemia, even after adjustment for personal medications.

Though bile is the major route of excretion, Hg can be reabsorbed into the blood via the enterohepatic system [12,32]. In particular, methylated Hg makes up most of the mercury in humans and can easily bind to cysteine residues [33], such as glutathione, and penetrate the cellular membranes [34]. The MeHg-cysteine complex can then enter the bile tract and be hydrolyzed by GGT and other dipeptides [13,33]. As a result, Hg induces hydrogen peroxide, depletes glutathione, and increases GGT levels. The association between Hg exposure and GGT, a marker of oxidative stress, is supported by several animal and human studies [35,36,37]. Our study also showed that the levels of blood Hg were significantly higher (*p* < 0.0001) in the elevated liver enzymes group (male: 4.36 μg/L, female: 3.25 μg/L) compared to the normal group (male: 3.64 μg/L, female: 2.70 μg/L). After adjustment for personal medications, blood Hg was associated with 35% higher odds of elevated liver enzymes.

This study has several limitations and strengths. The study design is a cross-sectional survey, and each measurement was analyzed from an individual spot sample. However, the dataset is representative of the entire Korean population. According to the previous studies, the intra-class correlation for blood Hg was 0.67~0.71 [38]. Moreover, diet is the major source of Hg, so we anticipate that the individual blood Hg levels would be constant. Secondly, individual health status or medical history data were unavailable. Instead, personal medication data were adjusted for the obesogenic effects of Hg. We also performed correlation analyses between the lipid profiles and hepatic enzymes to avoid overestimating the Hg effects. There were no associations between the lipid profiles and hepatic enzymes, nor were there any differences across blood Hg groups (Figure 2). This indicates that the effects of blood Hg on the lipid profiles were irrelevant to the hepatic enzymes and that the hepatic enzymes were not affected by the lipid profiles. Thus, correlation analyses demonstrate the significant effects of blood Hg on hyperlipidemia and elevated liver enzymes.

## 5. Conclusions

In this study, we investigated the obesogenic properties of blood Hg using lipid profiles and hepatic enzymes. Higher blood Hg levels were observed in the hyperlipidemia group than in the non-hyperlipidemia group, and the elevated liver enzymes group had higher mean blood Hg levels than the normal group. Blood Hg was associated with higher odds of hyperlipidemia and elevated liver enzymes, even after adjusting for personal medications. These results indicate that Hg exposure is associated with obesity-related outcomes and that other health effects due to low-level Hg exposure should be investigated.

## Figures and Tables

**Table 1:** Blood Hg distributions by demographic variables (μg/L).

Variables	N	GM	95 % Confidence Interval		P75	P95	*p*-Value *^a^*
All	6454	3.11	(3.02, 3.20)		4.69	9.01	-
Sex							<0.0001
Male	2767	3.70	(3.57, 3.84)		5.48	10.2	
Female	3687	2.63	(2.54, 2.72)		3.83	7.11	
Age							<0.0001
19–29	536	2.37	(2.23, 2.52)		3.22	6.93	
30–39	1053	3.18	(3.04, 3.33)		4.71	8.10	
40–49	1224	3.58	(3.43, 3.75)		5.25	9.56	
50–59	1434	3.64	(3.47, 3.82)		5.21	10.4	
60–69	1326	3.23	(3.05, 3.43)		4.85	9.17	
70+	881	2.54	(2.38, 2.71)		3.86	8.40	
BMI							<0.0001
<18.5	159	2.13	(1.85, 2.45)		2.76	5.61	
18.5 to < 23.0	2209	2.74	(2.64, 2.85)		4.08	7.61	
23.0 to < 25.0	1602	3.27	(3.13, 3.41)		4.90	9.05	
≥25.0	2484	3.54	(3.41, 3.68)		5.24	9.95	
Smoke							<0.0001
Non-smoker	4244	2.74	(2.65, 2.83)		4.03	7.62	
Past	1053	3.92	(3.71, 4.14)		5.82	11.0	
Current	1157	3.81	(3.64, 4.00)		5.71	10.03	
Alcohol frequency							<0.0001
Never	2219	2.68	(2.57, 2.79)		4.07	7.49	
<1 time/month	709	2.68	(2.53, 2.84)		3.78	6.90	
1–3 times/month	1033	2.94	(2.79, 3.09)		4.28	7.83	
1–2 times/week	1400	3.47	(3.30, 3.64)		5.25	9.67	
>3 times/week	612	4.03	(3.80, 4.27)		5.93	11.5	
Daily	481	4.08	(3.77, 4.43)		5.85	13.3	
Household income (USD/month)							<0.0001
<1500	1792	2.76	(2.62, 2.92)		4.29	8.98	
1500 to < 3000	1621	3.01	(2.84, 3.18)		4.52	8.79	
3000 to < 5000	1765	3.21	(3.08, 3.35)		4.70	7.91	
5000 to < 10,000	1103	3.37	(3.19, 3.56)		5.01	9.50	
≥10,000	173	3.47	(3.06, 3.92)		5.19	9.97	
Fish consumption frequency							<0.0001
Rarely	622	2.17	(2.03, 2.32)		3.07	6.95	
1–3 times/month	2030	2.88	(2.77, 2.99)		4.36	7.76	
1–3 times/week	3377	3.41	(3.30, 3.53)		5.03	9.37	
4–6 times/week	425	4.04	(3.68, 4.43)		6.20	11.2	

Note: GM, geometric mean; P75, 75^th^ percentile; P95, 95^th^ percentile. *^a^ p*-Value obtained from bivariate analysis (SAS Proc SURVEYREG).

**Table 2:** Lipid profiles and hepatic enzymes among the blood Hg groups.

Biomarkers (units)	Blood Hg	Male				Female				All				*p*-Value *^b^*
	Group *^a^*	N	GM	(P5	P95)	N	GM	(P5	P95)	N	GM	(P5	P95)	
**LDL** **(mg/dL)**	Low	613	82.7	(45.3	143)	1534	89.6	(52.1	144)	2156	87.3	(48.8	143)	0.0085
	Middle	895	84.5	(40.1	146)	1236	92.3	(53.8	150)	2142	88.4	(45.8	148)	
	High	1234	86.7	(40.1	146)	905	91.2	(50.0	152)	2156	88.2	(43.3	149)	
**HDL** **(mg/dL)**	Low	613	48.9	(33.2	73.6)	1534	56.0	(36.5	83.5)	2156	53.6	(35.0	80.6)	0.0012
	Middle	895	49.6	(33.6	72.8)	1236	56.4	(37.8	85.8)	2142	52.9	(34.7	80.5)	
	High	1234	49.2	(33.3	72.4)	905	55.7	(37.2	84.4)	2156	51.3	(34.1	77.1)	
**Total cholesterol** (**mg/dL)**	Low	613	171	(126	234)	1534	178	(129	240)	2156	176	(128	238)	<0.0001
	Middle	895	180	(129	244)	1236	183	(135	248)	2142	181	(132	246)	
	High	1234	184	(132	246)	905	186	(137	242)	2156	185	(134	245)	
**TG** **(mg/dL)**	Low	613	139	(49.6	370)	1534	119	(49.2	303)	2156	125	(49.4	331)	0.5822
	Middle	895	160	(63.2	496)	1236	127	(49.2	365)	2142	143	(56.5	415)	
	High	1234	176	(72.8	461)	905	134	(53.8	393)	2156	160	(62.6	437)	
**ALT** **(U/L)**	Low	613	22.2	(10.3	62.4)	1534	16.5	(8.54	33.8)	2156	18.2	(8.99	42.7)	0.0002
	Middle	895	25.5	(11.7	71.6)	1236	18.1	(9.39	38.2)	2142	21.5	(10.0	55.4)	
	High	1234	27.4	(12.6	69.7)	905	19.7	(9.93	45.0)	2156	24.5	(11.1	60.9)	
**AST** **(U/L)**	Low	613	24.0	(16.0	39.1)	1534	21.4	(14.1	32.9)	2156	22.2	(14.6	34.9)	0.0226
	Middle	895	25.8	(16.2	45.0)	1236	21.9	(15.0	34.2)	2142	23.8	(15.3	39.0)	
	High	1234	26.1	(16.7	46.0)	905	23.1	(15.0	36.5)	2156	25.0	(16.0	44.0)	
**GGT** **(U/L)**	Low	613	25.7	(11.1	106)	1534	15.9	(7.93	41.7)	2156	18.6	(8.36	56.5)	<0.0001
	Middle	895	32.6	(12.6	132)	1236	17.8	(8.60	51.5)	2142	24.0	(9.46	88.7)	
	High	1234	39.2	(13.9	152)	905	19.9	(9.04	58.2)	2156	31.1	(10.7	134)	

Note: GM, geometric mean; P5, 5^th^ percentile; P95, 95^th^ percentile. *^a^* Blood Hg was categorized into three groups - low: ≤ 2.36 μg/L, middle: 2.36 < Hg ≤ 4.07 μg/L, or high: > 4.07 μg/L. *^b^ p*-Values are the significance of blood Hg levels for each clinical marker, and the regression model included sex, age, BMI, smoking frequency, alcohol frequency, and income.

**Table 3:** The geometric means of blood Hg and the associations with hyperlipidemia (unit: μg/L).

Lipid Profiles		Criteria	Male		Female		*p*-Value *^a^*	*p*-Value *^b^*
			N	GM	N	GM		
LDL	<100	Optimal	1640	3.70	1982	2.70	0.0009	0.1054
	100–129	Above optimal	791	3.85	1161	2.84		
	130–159	Borderline high	285	4.31	434	2.80		
	160–189	High	40	4.19	95	3.13		
	≥190	Very high	11	4.05	15	2.79		
HDL	<40	Low	552	3.53	321	2.59	0.0004	0.1730
	40–60	Optimal	1658	3.87	2023	2.77		
	≥60	High	557	3.92	1343	2.82		
Total	<200	Desirable	1955	3.64	2504	2.71	<0.0001	<0.0001
cholesterol	200–239	Borderline high	654	4.28	909	2.86		
	≥240	High	158	4.13	274	2.95		
TG	<150	Normal	1286	3.59	2235	2.73	<0.0001	<0.0001
	150–199	Borderline high	519	4.01	599	2.71		
	200–499	High	854	4.00	794	2.94		
	≥500	Very high	107	4.02	58	2.63		
Hyperlipidemia *^c^*		No	1057	3.48	1698	2.69	<0.0001	<0.0001
		Yes	1710	4.03	1989	2.83		

Note: GM, geometric mean; LDL, low-density lipoprotein; HDL, high-density lipoprotein; TG, triglyceride. *^a^ p*-Value obtained using two-way ANOVA of the lipid profiles and sex. *^b^ p*-Value obtained by ANCOVA adjusted for age. *^c^* Hyperlipidemia was identified according to the following criteria - LDL ≥ 130, total cholesterol ≥ 200, or TG ≥ 150.

**Table 4:** The geometric means of blood Hg and the associations with elevated liver enzymes (unit: μg/L)**.**

Hepatic Enzymes	Male			Female			*p*-Value *^a^*	*p*-Value *^b^*
	Criteria	N	GM	Criteria	N	GM		
ALT	≤49	2513	3.78	≤49	3583	2.75	<0.0001	<0.0001
	>49	254	4.14	>49	104	3.56		
AST	<34	2373	3.76	<34	3446	2.74	<0.0001	<0.0001
	≥34	394	4.14	≥34	241	3.22		
GGT	<73	2361	3.67	<38	3305	2.71	<0.0001	<0.0001
	≥73	406	4.73	≥38	382	3.30		
Elevated	No	2096	3.64	No	3169	2.70	<0.0001	<0.0001
Liver enzymes *^c^*	Yes	671	4.38	Yes	518	3.25		

Note: GM, geometric mean; *^a^ p*-Value obtained by two-way ANOVA of the hepatic enzymes and sex. *^b^ p*-Value obtained by ANCOVA adjusted for age. *^c^* Elevated liver enzymes were identified according to the following criteria - ALT > 49, AST ≥ 34, or GGT ≥ 73 for males, and ALT > 49, AST ≥ 34, or GGT ≥ 38 for females.

**Table 5:** The relationships between blood Hg and hyperlipidemia and elevated liver enzymes.

Disease	Personal Medication *^a^*	GM	OR	95 % CI	*p*-Value *^b^*
Hyperlipidemia (*n* = 3699)					
	Unadjusted	3.33	1.105	(1.013, 1.206)	0.0252
	Hyperlipidemia	3.12	1.104	(1.012, 1.206)	0.0266
	Hyperlipidemia and Hypertension	2.95	1.104	(1.011, 1.205)	0.0275
	Hyperlipidemia and Diabetes	3.60	1.105	(1.013, 1.207)	0.0250
	Hyperlipidemia and Hypertension and Diabetes	3.16	1.104	(1.012, 1.206)	0.0263
	One of the Hyperlipidemia, Hypertension, Diabetes	3.19	1.100	(1.007, 1.201)	0.0335
Elevated liver enzymes (*n* = 1189)					
	Unadjusted	3.84	1.345	(1.206, 1.500)	<0.0001
	Liver disease	3.24	1.350	(1.210, 1.506)	<0.0001

Note: GM, geometric mean; OR, odds ratio; 95% CI, 95% confidence interval. *^a^* Personal medication information was included in the model from the self-reported response. *^b^ p*-Value shows the significance of the odds ratio of blood Hg from logistic regression. The unadjusted model included the main effects of sex, age, BMI, smoke, alcohol frequency, fish consumption, and the two-way interaction of sex and alcohol frequency. Each personal medication information was included in the unadjusted model.

## Supplementary Materials

The following are available online at https://www.mdpi.com/2305-6304/8/3/47/s1, Table S1: Blood Hg distributions by influential variables.
