# Supplementary material for: Mercury Exposure and Associations with Hyperlipidemia and Elevated Liver Enzymes: A Nationwide Cross-Sectional Survey
Source: Toxics. 2020 Jul 1;8(3):47. doi: 10.3390/toxics8030047 (PMC7560241; doi:10.3390/toxics8030047)
Supplement: Supplementary file 1 [file toxics-08-00047-s001.pdf]

Supplementary Materials: Mercury Exposure and Associations with Hyperlipidemia and Elevated Liver Enzymes: A Nationwide Cross-Sectional Survey

Seungho Lee, Sung-Ran Cho, Inchul Jeong, Jae Bum Park, Mi-Yeon Shin, Sungkyoon Kim and Jin Hee Kim

Table S1. Blood Hg distributions by influential variables.

Variables	N	GM	95% Confidence Interval	P75	P95	*p*-Value[a]
**All**	**6454**	**3.11**	**(3.02, 3.20)**	**4.69**	**9.01**	
Alcohol consumption amount						
Never	2219	2.68	(2.57, 2.79)	4.07	7.49	<0.0001
1~2 shots	1190	2.96	(2.79, 3.14)	4.33	7.82	
3~4 shots	987	3.11	(2.94, 3.29)	4.57	8.34	
5~6 shots	514	3.29	(3.08, 3.51)	4.71	9.22	
7~9 shots	782	3.60	(3.41, 3.81)	5.51	9.58	
10 or over shots	762	3.71	(3.50, 3.94)	5.65	11.6	
Cooking types						
Rarely	485	2.15	(1.97, 2.34)	3.08	7.14	<0.0001
Baking/Roasting	2488	3.15	(3.03, 3.28)	4.69	8.58	
Steaming/Boiling/Stewing	1647	3.23	(3.09, 3.38)	4.85	9.48	
Stir-fry/Sauteing	1641	3.16	(3.01, 3.31)	4.65	8.64	
Raw	193	4.18	(3.70, 4.74)	6.29	11.6	
Herbal medicine						
No	5719	3.12	(3.02, 3.21)	4.71	9.05	0.6199
Yes	735	3.07	(2.88, 3.27)	4.52	8.85	
Marital status						
Single	654	2.44	(2.30, 2.59)	3.41	6.93	<0.0001
Married	5109	3.36	(3.26, 3.46)	4.95	9.36	
Divorce/Separation/Bereavement	691	2.71	(2.50, 2.93)	4.05	9.62	
Parity (only female) [b]						
No	390	2.15	(2.01, 2.29)	2.81	5.82	<0.0001
Yes	3295	2.75	(2.66, 2.85)	4.04	7.31	
Menopause (only female)						
No	1669	2.55	(2.45, 2.65)	3.60	6.83	0.0047
Yes	2016	2.74	(2.62, 2.87)	4.13	7.79	
Education						
Never	2283	2.91	(2.76, 3.06)	4.44	8.64	<0.0001
Elementary school	1943	3.30	(3.17, 3.43)	4.88	9.22	
Middle school	2017	3.06	(2.94, 3.19)	4.69	8.97	
High school or over	200	3.49	(3.16, 3.87)	5.03	10.94	

Note: GM, geometric mean; P75, 75 percentile; P95, 95 percentile. [a]*p*-Value obtained using bivariate analysis (SAS Proc SURVEYREG). [b]Parity was defined as whether a women gave birth or not. Thus, 'yes' indicates that she gave birth to a baby, and 'no' indicates that she has never given birth.
